# Impact of age on adjuvant chemotherapy after radical resection in patients with non–small cell lung cancer

**DOI:** 10.1002/cam4.814

**Published:** 2016-07-01

**Authors:** Xiaoyu Zhai, Lu Yang, Sipeng Chen, Qiwen Zheng, Ziping Wang

**Affiliations:** ^1^ Medical Oncology DepartmentCancer HospitalChinese Academy of Medical SciencesPeking Union Medical CollegeBeijing100021China; ^2^ Department of Epidemiology and Health StatisticsCapital Medical University School of Public HealthBeijing100053China; ^3^ Medical Insurance OfficePeking University Cancer Hospital & InstituteBeijing100142China

**Keywords:** Adjuvant chemotherapy, age, non–small cell lung cancer (NSCLC)

## Abstract

Adjuvant chemotherapy (ACT) after radical surgery is known to improve the survival of patients with non–small cell lung cancer (NSCLC). However, there are few studies reporting the impact of age on the efficacy of ACT in NSCLC patients. All patients who received postoperative ACT in the Cancer Hospital, the Chinese Academy of Medical Sciences, between 2001 and 2013 with complete records in the database of the hospital and met the inclusion criteria were enrolled in this study for analysis. The primary end point was disease‐free survival (DFS) in terms of age. Survival analysis was performed using Kaplan–Meier estimates, log‐rank tests, and Cox's proportional hazards regression analysis. Propensity score matching (PSM) was used, survival analysis and subgroup analysis of the match data were carried out. Of 1095 patients with stage IB to stage IIIA NSCLC who underwent radical resection, 865 cases who met the inclusion criteria were analyzed. Of them, 156 (18.0%) patients were 65 years old or older, and the remaining 709 (82.0%) patients were younger than 65. The DFS between the younger group and the elderly group was not significantly different neither before PSM (100.714 weeks [95% CI: 84.421, 117.007] vs. 99.571 weeks [95% CI: 82.621, 116.522]; *P *= 0.555) nor after PSM (104.857 weeks [95% CI: 81.495, 128.220] vs. 97.429 weeks [95% CI: 81.743, 113.114]; *P *= 0.328) using the Kaplan–Meier method.The results suggest that the benefit on DFS was similar regardless of age of NSCLC patients. ACT should not be withheld from elderly patients. However, these conclusions are limited by the nature of this retrospective study, and therefore prospective trials are required for further verification.

## Introduction

Lung cancer is the most common malignancy worldwide 1, and seems to be a disease of the elderly 2 because approximately 50% lung cancer cases were diagnosed in patients older than 70 years 3. The prevalence and social burden of the disease are expected to further increase as more people survive into old age.

Elderly cancer patients are obviously underrepresented in most clinical trials, including those with lung cancer 4, 5, 6. There are controversies over how to perform postoperative adjuvant chemotherapy (ACT) in elderly patients with non–small cell lung cancer (NSCLC). A meta‐analysis involving 3324 patients older than 65 years showed that platinum‐based ACT was associated with reduced mortality and increased risk of serious adverse events in the patient group with stages II–IIIA NSCLC 7. Another study reported that ACT offered a significant benefit of survival in 155 NSCLC patients older than 65 years 8. Still another report concluded that elderly patients derived a similar magnitude of benefit from ACT as younger patients 9. Based these studies, researchers appeal that ACT should not be withheld from elderly patients 8, 9, 10.

However, there is substantial underrepresentation of patients aged more than 65 years in clinical trials concerning malignancies 11. Furthermore, treatment decisions are based on results of trials conducted in relatively younger individuals. Besides, the conclusion that aged patients should not be withheld from ACT only by age is mainly based on subgroup analysis in most retrospective studies 12, 13, 14. In other words, most of these analyses did not take into consideration important clinical factors that might bias the result. Thus, the conclusive evaluations of these trials are less objective. The aim of this study was to evaluate the efficacy of ACT in elderly NSCLC patients more objectively by comparing them with the younger group using propensity score matching (PSM).

## Methods

### Patients

The study was approved by the Ethics Committee of the Cancer Hospital of the Chinese Academy of Medical Sciences (Beijing, China). A total of 1095 patients who had received ACT after radical resection in the said hospital between January 2001 and December 2013 were enrolled in this study. After excluding some patients: those with stage IB NSCLC without high‐risk factors, those with stage IIIB NSCLC or multiple primary malignant tumors, those who had received preoperative radiotherapy or chemotherapy, those used changed regimen of ACT, and those with uncertain T staging. A total of 865 patients met our inclusion criteria and the data were analyzed. The last follow up was carried by the end of October 2015. Data obtained from hospital computer information systems, letters, follow‐up scans, and telephone calls, and the information were collected into our database for analysis.

The indication for ACT was in accordance with the latest edition of the NCCN guidelines in terms of pathologic findings. All staging procedures were carried out using the 7th edition of the American Joint Committee on Cancer (AJCC) Cancer Staging Manual. The Charlson Comorbidities index (CCI) was defined according to the 10th revision of the International Classification of Diseases (ICD)–10 diagnosis codes 15. And the comorbidities occurred before diagnosing was carefully recorded. The performance status (PS) was recorded according to the Eastern Cooperative Oncology Group (ECOG) performance scale. Disease‐free survival (DFS) was defined as the length of time from lung cancer resection to any kind of recurrence. Recurrences were classified as three degrees: local recurrence (LR), which was defined as any recurrence occurring on resection margins such as bronchial stumps or stapler lines; regional recurrence (RR), which was defined as any recurrence occurring in the hilus or mediastinal lymph node, pleural cavity, and ipsilateral lung; and distant recurrence (DR), which was defined as any recurrence occurring in the contralateral lung, brain, liver, adrenal gland, bone, and other locations. Disease recurrence was assessed by computed tomography (CT) scan, magnetic resonance imaging (MRI), bone scanning, and tumor markers.

### Statistical analysis

Data are presented as number (%) and hazard ration (HR) (95% confidence interval CI). Baseline data are presented as mean ± SD continuous variables, and as frequencies and percentages for categorical variables. Continuous variables were compared using t tests, and categorical variables were compared using *χ* ^2^ tests. The baseline characteristics included gender, smoking history, PS, CCI, histological subtype, differentiation, T and N stage, clinical stage, type of surgical procedures, regimen and number of cycles of adjuvant chemotherapy, time to adjuvant chemotherapy (TTAC), and adjuvant radiotherapy.

The primary end point was disease‐free survival (DFS). Survival curves were analyzed by Kaplan–Meier methods. Univariate analyses were performed by using the log‐rank test. The multivariable analysis was performed by Cox proportional hazard regression model. Histological subtype, differentiation, T and N stage, type of surgical procedures, regimen and number of cycles of adjuvant chemotherapy, adjuvant radiotherapy, and age were included as variables.

To overcome the impact of bias and potential confounding factors in the retrospective study, we performed rigorous adjustment for baseline differences by PSM. To estimate the propensity score, a logistic regression model predicting the age was developed using the covariates. For each comparison, a separate propensity score for age was derived. For matching, the elderly and otherwise pair with an equivalent propensity score was selected by a 1‐to‐3 match. In the PSM cohort, the risk of each outcome was compared using Cox regression models.

All reported *P*‐values are two sided, and *P*‐values of more than 0.05 were considered to indicate statistical significance. All statistical analyses were performed using SAS 9.3 (SAS Institute Inc., Cary, NC).

## Results

Patients who received postoperative ACT in the Cancer Hospital of the Chinese Academy of Medical Sciences between 2001 and 2013 were included in our retrospective study. A total of 156 (18.0%) patients were 65 years and older, and 709 (82.0%) NSCLC patients younger than 65 years old. The clinical characteristics of patients before and after PSM are summarized in Table 1. The basic characteristics were balanced between the different age group in terms of smoking history, PS, histological subtype, T and N stage, clinical stage, regimen, and number of cycles of adjuvant chemotherapy prior to PSM.

**Table 1:** Demographic and clinical characteristics of patients with Stage IB to IIIA NSCLC according to age before and after propensity score matching

Characteristic	Before propensity score matching			After propensity score matching		
	Age <65*N *= 709(%)	Age ≥65*N *= 156(%)	*P* value	Age <65 *N *= 387(%)	Age ≥65*N* = 129(%)	*P* value
Gender						
Male	445 (62.8)	116 (74.4)	0.006	271 (70.0)	92 (71.3)	0.781
Female	264 (37.2)	40 (25.6)		116 (30.0)	37 (28.7)	
Smoking history						
No	334 (47.1)	65 (41.7)	0.217	166 (42.9)	58 (45.0)	0.682
Yes	375 (52.9)	91 (58.3)		221 (57.1)	71 (55.0)	
PS						
0	138 (19.5)	25 (16.0)	0.320	62 (16.0)	22 (17.1)	0.783
≥1	571 (80.5)	131 (84.0)		325 (84.0)	107 (82.9)	
Charlson comorbidities index						
0	529 (74.6)	102 (65.4)	0.037	265 (68.5)	90 (69.8)	0.951
1	132 (18.6)	36 (23.1)		83 (21.4)	26 (20.2)	
≥2	48 (6.8)	18 (11.5)		39 (10.1)	13 (10.1)	
Histological subtype						
Squamous	210 (29.6)	43 (27.6)	0.409	109 (28.2)	33 (25.6)	0.515
Adenocarcinoma	472 (66.6)	110 (70.5)		266 (68.7)	94 (72.9)	
Other	27 (3.8)	3 (1.9)		12 (3.1)	2 (1.6)	
Differentiation						
Poorly	153 (21.6)	47 (30.1)	0.016	100 (25.8)	35 (27.1)	0.109
Moderately	513 (72.4)	101 (64.7)		270 (69.8)	86 (66.7)	
Well	25 (3.5)	1 (0.6)		10 (2.6)	1 (0.8)	
Unknown	18 (2.5)	7 (4.5)		7 (1.8)	7 (5.4)	
T stage						
T1A	51 (7.2)	7 (4.5)	0.112	33 (8.5)	6 (4.7)	0.204
T1B	53 (7.5)	4 (2.6)		22 (5.7)	4 (3.1)	
T2A	448 (63.2)	104 (66.7)		234 (60.5)	91 (70.5)	
T2B	59 (8.3)	13 (8.3)		31 (8.0)	12 (9.3)	
T3	69 (9.7)	17 (10.9)		46 (11.9)	13 (10.1)	
T4	29 (4.1)	11 (7.1)		21 (5.4)	3 (2.3)	
N stage						
N0	160 (22.6)	42 (26.9)	0.329	102 (26.4)	30 (23.3)	0.178
N1	224 (31.6)	52 (33.3)		102 (26.4)	45 (34.9)	
N2	325 (45.8)	62 (39.7)		183 (47.3)	54 (41.9)	
Clinical stage						
IB	123 (17.3)	22 (14.1)	0.236	77 (19.9)	20 (15.5)	0.085
IIA	185 (26.1)	42 (26.9)		72 (18.6)	37 (28.7)	
IIB	36 (5.1)	14 (9.0)		26 (6.7)	10 (7.8)	
IIIA	365 (51.5)	78 (50.0)		212 (54.8)	62 (48.1)	
Type of surgical procedures						
Lobectomy	639 (90.1)	148 (94.9)	0.028	352 (91.0)	120 (93.0)	0.073
Pneumonectomy	64 (9.0)	5 (3.2)		33 (8.5)	6 (4.7)	
Others	6 (0.8)	3 (1.9)		2 (0.5)	3 (2.3)	
No. of adjuvant chemotherapy cycles						
<4	70 (9.9)	23 (14.7)	0.206	39 (10.1)	21 (16.3)	0.142
=4	610 (86.0)	127 (81.4)		333 (86.0)	102 (79.1)	
>4	29 (4.1)	6 (3.8)		15 (3.9)	6 (4.7)	
Regimen						
Pemetrexed	166 (23.4)	32 (20.5)	0.054	103 (26.6)	27 (20.9)	0.220
Paclitaxel	236 (33.3)	72 (46.2)		137 (35.4)	58 (45.0)	
Gemcitabine	187 (26.4)	31 (19.9)		94 (24.3)	24 (18.6)	
Vinorelbine	96 (13.5)	17 (10.9)		46 (11.9)	16 (12.4)	
Docetaxel	24 (3.4)	4 (2.6)		7 (1.8)	4 (3.1)	
Time to adjuvant chemotherapy						
<4w	106 (15.0)	13 (8.3)	0.003	38 (9.8)	13 (10.1)	0.602
4–6w	437 (61.6)	88 (56.4)		246 (63.6)	76 (58.9)	
>6w	166 (23.4)	55 (35.3)		103 (26.6)	40 (31.0)	
Adjuvant radiotherapy						
No	592 (83.5)	142 (91.0)	0.018	348 (89.9)	115 (89.1)	0.867
Yes	117 (16.5)	14 (9.0)		39 (10.1)	14 (10.9)	

The results of Cox proportional hazard model analysis are shown in Table 2. Factors that predicted significantly improved DFS included squamous carcinoma, lower N stage, and adjuvant radiotherapy. The result of analysis stratified by histological differentiation showed that the NSCLC patients with moderately differentiated carcinoma were associated with improved DFS comparing with the poor subgroup (HR: 0.685, [95% CI: 0.557–0.843]; *P* < 0.001). And the patients who are successful in fulfilling four cycles of adjuvant chemotherapy got better DFS comparing with those with less than four cycles (HR: 0.727, [95% CI: 0.552–0.958]; *P *= 0.023). Conversely, neither paclitaxel regimen (HR: 1.331, [95% CI: 1.026–1.728]; *P* = 0.031) nor docetaxel regimen (HR: 2.100, [95% CI: 1.305–3.378]; *P* = 0.002) were associated with improved DFS. As for the type of surgical procedures, the NSCLC patients who has undergone pneumonectomy (HR: 1.478, [95% CI: 1.073–2.035]; *P* = 0.017) achieved worse DFS comparing with lobectomy. Besides, T3 stage (HR: 1.574, [95% CI: 1.025–2.417]; *P* = 0.038) was associated with worse DFS comparing with T1A patients.

**Table 2:** Cox analysis of stages IB to IIIA NSCLC patients receiving adjuvant chemotherapy after radical resection

Characteristic		HR (95% CI)	*P*
Histological subtype	Adenocarcinoma versus squamous	1.515 (1.199–1.916)	0.001
	Other versus squamous	2.172 (1.316–3.585)	0.002
Differentiation	Moderately versus poorly	0.685 (0.557–0.843)	<0.001
	Well versus poorly	0.684 (0.363–1.287	0.239
	Unknown versus poorly	0.832 (0.449–1.543)	0.560
T stage	IB versus IA	0.809 (0.497–1.318)	0.395
	IIA versus IA	0.944 (0.665–1.340)	0.747
	IIB versus IA	1.258 (0.801–1.975)	0.319
	III versus IA	1.574 (1.025–2.417)	0.038
	IV versus IA	1.461 (0.836–2.556)	0.183
N stage	N1 versus N0	2.507 (1.836–3.424)	<0.001
	N2 versus N0	5.055 (3.746–5.820)	<0.001
Type of surgical procedures	Pneumonectomy versus lobectomy	1.478 (1.073–2.035)	0.017
	Other versus lobectomy	1.532 (0.627–3.743	0.349
Regimen	Paclitaxel versus pemetrexed	1.331 (1.026–1.728)	0.031
	Gemcitabine versus pemetrexed	1.179 (0.872–1.595)	0.285
	Vinorelbine versus pemetrexed	1.175 (0.855–1.614)	0.320
	Docetaxel versus pemetrexed	2.100 (1.305–3.378)	0.002
No. of adjuvant chemotherapy cycles	=4 versus <4	0.727 (0.552–0.958)	0.023
	>4 versus <4	0.812 (0.498–1.323)	0.403
Adjuvant radiotherapy		0.727 (0.565–0.935)	0.013
Age		1.022 (0.812–1.285)	0.856

**Figure 1:**
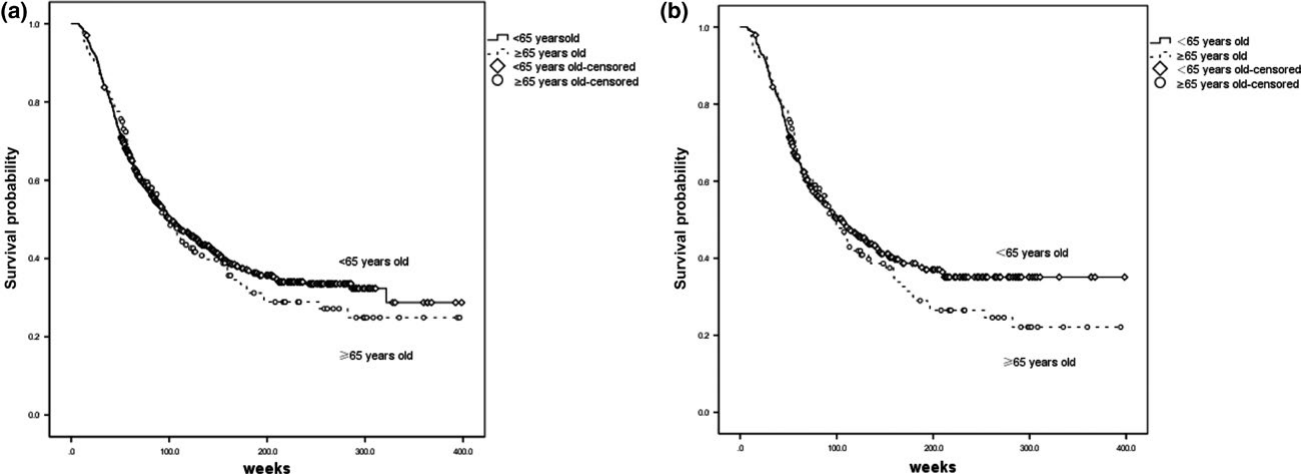
Kaplan–Meier Plot of disease‐free survival (DFS) by age.

**Figure 2:**
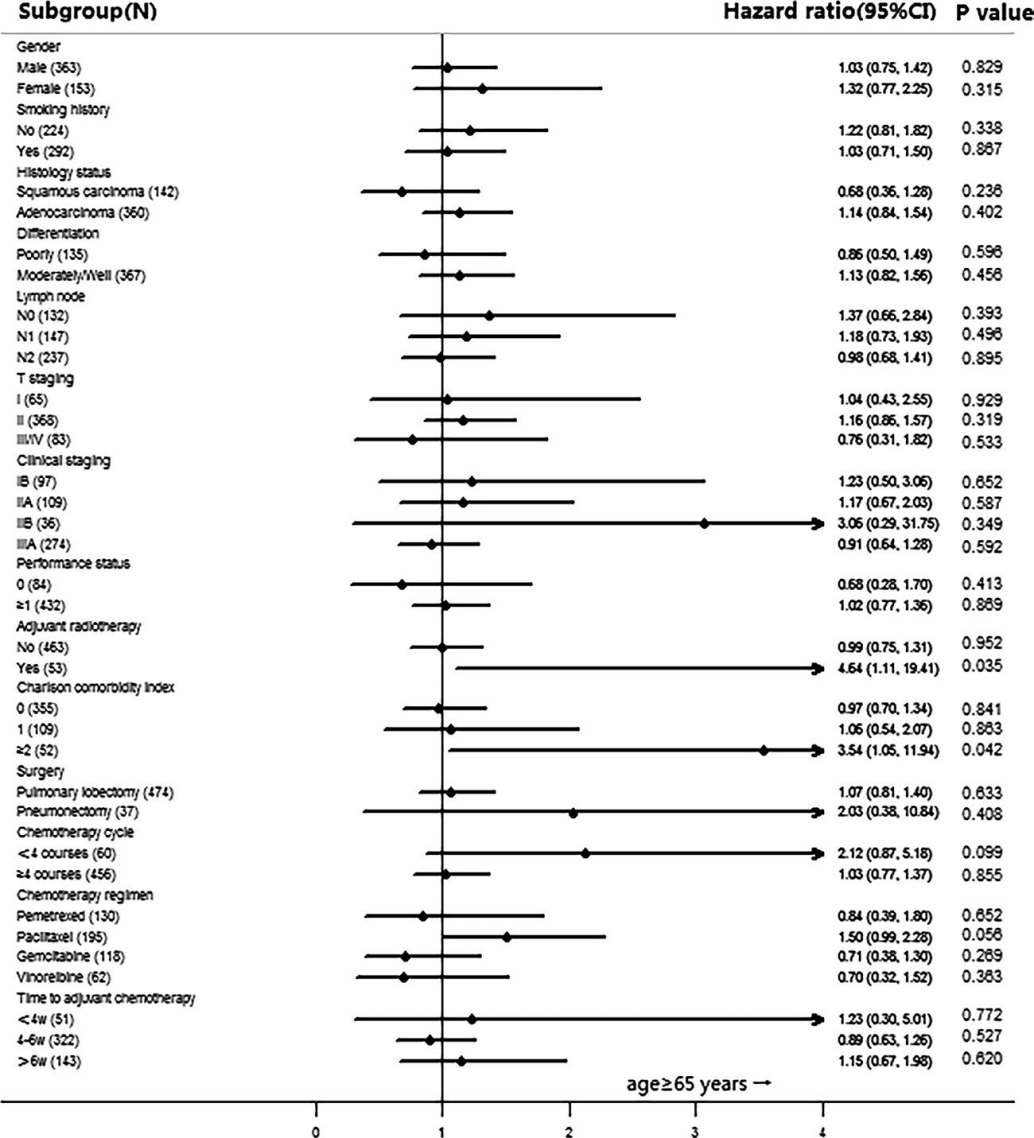
Forest Tree Plot of Hazard Ratio.

Finally, 387 patients younger than 65 years and 129 elderly patients were matched, and the baseline characteristics were well balanced after the PS matching process. The survival curves in terms of age before and after PSM are shown in Figure 1. The DFS between the younger group and the elderly group was not significantly different neither before PSM (100.714 weeks [95% CI: 84.421, 117.007] vs. 99.571 weeks [95% CI: 82.621, 116.522]; *P* = 0.555) nor after PSM (104.857 weeks [95% CI: 81.495, 128.220] vs. 97.429 weeks [95% CI: 81.743, 113.114]; *P* = 0.328) using the Kaplan–Meier method. And for subgroup of NSCLC patients who received adjuvant radiotherapy, the analysis showed that the benefit is in favor of younger group of patients comparing with elderly patients in terms of DFS (HR: 4.64 [95% CI: 1.11–19.41]; *P* = 0.035). There was still significant less DFS in groups of patients with two or more comorbidities (HR: 3.54 [95% CI: 1.05–11.94]; *P* = 0.042) (Fig. 2).

## Discussion

What is the optimal ACT for elderly NSCLC patients remains controversial because of lacking prospective study. To the best of our knowledge, the only prospective clinical trial conducted in NSCLC patients older than 65 years who received ACT is concerned with Health‐Related Quality of Life (HRQOL), saying that postoperative chemotherapy did not substantially reduce HRQOL in elderly NSCLC patients 16. However, this study did not concern the survival difference between older and younger NSCLC patients. Clinical trials open to subjects of any age reported that elderly patients could benefit from postoperative ACT 17. However, fewer than 10% patients enrolled in these trials were older than 70 years and therefore the clinical value of this conclusion is limited. In this study, we sought to evaluate the efficacy of ACT between elderly and the younger patients more objectively in terms of DFS by using PSM.

Benefit achieved on ACT in the aged patients mainly came from retrospective studies or population observation studies. Few studies have focused on analysis of differences in ACT‐related benefits between different age groups. Our analyses indicate that DFS was similar between the two groups of NSCLC patients whether or not PSM, which is consistent with some previous studies 7, 8, 9, 10, 18.

The National Cancer Institute of Canada Clinical Trials Group JBR.10 (NCIC‐CTG JBR.10) trial concerning adjuvant chemotherapy involved in 327 young (≤65 years) and 155 elderly (>65 years) NSCLC patients. The subgroup analysis of this study showed that OS was similar between younger patients and elderly subgroup before 75‐year age 8. One subgroup analysis of the Lung Adjuvant Cisplatin Evaluation (LACE) study indicated that there was no significant difference in either OS or event‐free survival (EFS) between old‐age group (≥70 years), middle‐age group (65–69 years), and younger‐age group (<65 years) 10. However, LACE study and JBR.10 trial did not take more advanced statistical techniques to control selection bias when they compared the outcomes by age. A recent population‐based retrospective study including 2897 stage IB to stage III NSCLC patients older than 70 years who underwent surgical resection showed that the risk of death was reduced in both younger patients (HR: 0.79; 95% CI: 0.72–0.86) and elderly patients (HR: 0.81; 95% CI: 0.71–0.92) who received ACT, suggesting that elderly patients should not be withheld from ACT 9. Besides, some population‐based observational studies concluded that ACT was beneficial to elderly NSCLC patients 7, 18.

One Surveillance, Epidemiology, and End Results (SEER)–Medicare database analysis involving 3759 older than 65 years NSCLC patients analysis showed that both OS and the 5‐year adjusted survival rate in patients who received ACT were significantly better than those in patients without receiving ACT (HR: 0.80; 95% CI: 0.72 to 0.89 and 35% [95% CI: 32–39%] vs. 27% [95% CI: 25–29%]) 7. Cuffe et al. reviewed the survival of elderly patients between the period from 2001 to 2006, and found that the 4‐year survival rate in patients aged ≥70 years was increased significantly because of increased population who intake the adjuvant chemotherapy (47.1% in the period between 2001 and 2003 vs. 49.9% in the period between 2004 and 2006, *P* = 0.01)^18^. All these studies enlightened that elderly patients should not be withheld from ACT merely because of age. However, few investigators used PSM to explore the impact of age on the extent of survival benefit from postoperative ACT. The result of our study showed that as younger patients, elderly patients could also benefit from ACT.

Our study has some limitations as well. First, this is a retrospective study and all data were obtained from Chinese patients in a single medical center. Second, the adverse reaction was not inferred, in that some patients accomplished their further cycles of ACT in other hospital. In addition, knowing that EGFR mutation and ALK rearrangement in a large population of the Asian, personalized therapy and traditional Chinese medicine in their further line treatment could greatly affect OS; DFS has to be chosen as the primary end point in this study. Despite these limitations, this study has some unique advantages. Using PSM to greatly balance the important clinical characteristics, which we believe can control the bias to the greatest extent. Besides, as all our data are authentic and nonselective, they can represent the real world in clinical practice.

Lastly, in subgroup analysis of PS‐matched data showed that there was significant difference in elderly patients who were with two or more comorbidity scores (HR: 3.54 [95% CI: 1.05–11.94]; *P* = 0.042) or adjuvant radiotherapy (HR: 4.64 [95% CI: 1.11–19.41]; *P* = 0.035). There were sufficient analysis concluded that increased CCI score was associated with worse survival 19, 20. One analysis including 1,255 NSCLC patients showed that elderly patients were significantly associated with greater CCI comparing with younger patients (42% vs. 26%; *P* < 0.0001), and the overall survival was decreased in patients with CCI≥1 (HR: 1.28 [95% CI: 1.09–1.5]; *P* = 0.003) 20. To date, only one study involving 1307 patients aged ≥65 years who underwent radically resected NSCLC demonstrated that postoperative radiotherapy was not associated with improved survival in elderly patients with N2 disease (HR: 1.11; 95% CI: 0.97–1.27) 21. None of the study analysis the impact of age on the efficacy of postoperative radiotherapy. However, we cannot infer the conclusion for these two aspects because of insufficient sample size in subgroup analysis, and larger sample prospective studies are required to verify our observation.

## Conclusion

The results of our analysis indicate that DFS was similar between elderly and younger NSCLC patients. Elderly patients should not be withheld from ACT only by age. Elderly patients with more comorbidity scores and adjuvant radiotherapy might not benefit from their adjuvant chemotherapy and radiotherapy.

## Conflict of Interest

None.
